# 977. Impact of Chlorhexidine (CHG) vaginal preparation compared to betadine on rates of Total abdominal Hysterectomy (TAH) Surgical Site Infections (SSI)

**DOI:** 10.1093/ofid/ofad500.032

**Published:** 2023-11-27

**Authors:** Anupama Neelakanta, Jubilee Brown, Daniel Handel, Kristin Fischer, Catherine Passaretti

**Affiliations:** Atrium Health, Charlotte, NC; Atrium Health, Charlotte, NC; Atrium Health, Charlotte, NC; Atrium Health, Charlotte, NC; Advocate Health, Charlotte, NC

## Abstract

**Background:**

American College of Obstetricians and Gynecologists (ACOG) and the Centers for Disease Control (CDC) both recommend vaginal cleaning prior to surgery as part of hysterectomy SSI prevention. However, there is no clear guidance on preference of agent. Povidone-iodine is FDA approved for vaginal prep, but 4% chlorhexidine gluconate (CHG) is often used by surgeons as an acceptable alternative (off label) as there is some limited data to show improved bacterial counts with CHG.

**Methods:**

In 2020, our healthcare system implemented a vaginal hysterectomy SSI bundle consisting of preoperative CHG bathing day of surgery, alcohol containing skin preparation, vaginal preparation with betadine, or CHG per surgeon preference and adherence to system guidelines on preoperative antibiotics. Additional bundle components for open procedures included use of a separate closing tray, re-gowning/re-gloving and re-draping prior to closing. The TAH Bundle was not mandatory but highly recommended. Compliance was monitored and feedback was given to key stakeholders monthly. In the third quarter 2022, the bundle was modified to make CHG the preferred agent for vaginal prep.

We performed a retrospective study of patients who underwent inpatient and outpatient TAH between 1/1/2020 to 12/31/2022 comparing rates of SSIs between patients who received CHG vs betadine for vaginal preparation.

**Results:**

7327 TAH procedures were performed during the study period with rate of 1.24 TAH SSI per 100 procedures(Table 1). The rate of TAH SSI was 35% lower in the group receiving CHG vaginal prep compared to those who received betadine (IRR 0.65, p = 0.06). SSI rates and compliance with all components of the bundle varied by facility. The decrease associated with CHG vaginal prep compared to betadine was primarily in the group where all other bundle elements were followed (IRR 0.53, p=0.02) (Table 2).

**Figure:**
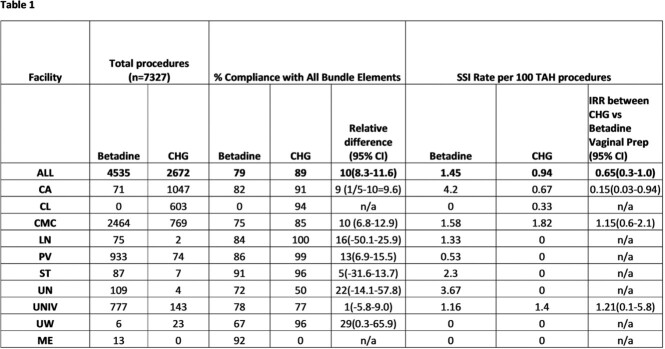

**Figure:**
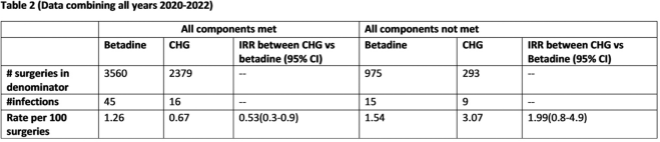

**Conclusion:**

In aggregate, use of CHG for vaginal prep was associated with a trend towards lower SSI. Use of CHG vaginal prep was associated with higher compliance with other bundle elements and facilities with higher compliance generally had lower SSI rates supporting bundle concept in SSI prevention. Limitations of this study include that individual patient level factors/case complexity, facility size were not taken into consideration.

**Disclosures:**

**All Authors**: No reported disclosures

